# New and Emerging Oral/Topical Small-Molecule Treatments for Psoriasis

**DOI:** 10.3390/pharmaceutics16020239

**Published:** 2024-02-06

**Authors:** Elena Carmona-Rocha, Lluís Rusiñol, Lluís Puig

**Affiliations:** 1 Department of Dermatology, Hospital de la Santa Creu i Sant Pau, 08041 Barcelona, Spain; ecarmona@santpau.cat (E.C.-R.); lrusinol@santpau.cat (L.R.); 2 Institut de Recerca Sant Pau (IR SANT PAU), 08041 Barcelona, Spain; 3 Sant Pau Teaching Unit, School of Medicine, Universitat Autònoma de Barcelona, 08041 Barcelona, Spain

**Keywords:** psoriasis, oral therapies, topical therapies, biologics, Jak inhibitors, PDE4 inhibitors, systemic treatment, IL-17 inhibitors, IL-23 inhibitors, tapinarof, roflumilast

## Abstract

The introduction of biologic therapies has led to dramatic improvements in the management of moderate-to-severe psoriasis. Even though the efficacy and safety of the newer biologic agents are difficult to match, oral administration is considered an important advantage by many patients. Current research is focused on the development of oral therapies with improved efficacy and safety compared with available alternatives, as exemplified by deucravacitinib, the first oral allosteric Tyk2 inhibitor approved for the treatment of moderate to severe psoriasis in adults. Recent advances in our knowledge of psoriasis pathogenesis have also led to the development of targeted topical molecules, mostly focused on intracellular signaling pathways such as AhR, PDE-4, and Jak-STAT. Tapinarof (an AhR modulator) and roflumilast (a PDE-4 inhibitor) have exhibited favorable efficacy and safety outcomes and have been approved by the FDA for the topical treatment of plaque psoriasis. This revision focuses on the most recent oral and topical therapies available for psoriasis, especially those that are currently under evaluation and development for the treatment of psoriasis.

## 1. Introduction

Psoriasis is a chronic inflammatory disease with a worldwide prevalence of 1–3% that can be associated with psoriatic arthritis and other comorbidities and causes a significant burden and impairment of health-related quality of life [1]. Psoriasis is an immune-mediated inflammatory disease (IMID) with a pathogenic admixture of autoinflammatory and autoimmune components and involvement of both innate and adaptive immune systems [2]. The success of biologic treatment in moderate to severe psoriasis has led to the development of multiple currently available alternatives, but subcutaneous administration may be inconvenient for certain patients. Several oral and topical treatments are currently being developed, including phosphodiesterase (PDE) 4 inhibitors, Janus kinase (Jak) inhibitors, and oral Interleukin (IL)-17 inhibitors, among others. The development of these novel therapies is a good example of applied translational research based on significant advances in our understanding of the immunopathogenesis of psoriasis, resulting in a wide therapeutic armamentarium with diverse mechanisms of action.

**Figure 1:**
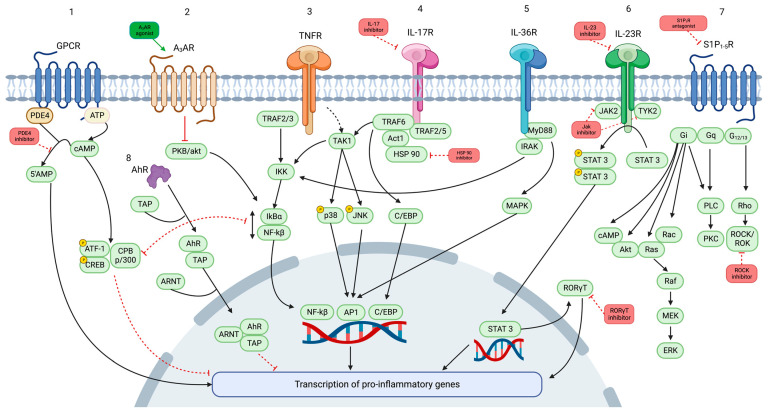
Main inflammatory pathways involved in psoriasis pathogenesis and current and potential therapeutic targets. Adapted from Rusiñol et al. [3]. (1) PDE4 inhibition increases intracellular levels of cAMP, activating PKA-mediated phosphorylation of the transcription factors CREB and ATF-1; this results in increased gene expression of anti-inflammatory cytokines and inhibition of NF-κB due to interaction with the coactivators CBP and p300. (2) Binding of A3AR agonist to its receptor (A3AR) inhibits the activity of PKB/Akt and reduces NF-κB activity. (3) Binding of TNF to TNFR activates the complex of TAK1 with its binding proteins; TAK1 phosphorylates the IKK complex and MAPKs JNK and p38, leading to activation of NF-κB and AP1, respectively, and proinflammatory gene expression. (4) Activation of the IL-17 receptor complex by IL-17A/F leads to recruitment of Act1, followed by TRAFs. TRAF6 activates the TAK1 pathway, resulting in activation of NF-κB and AP1. Inhibition of the chaperone HSP90 leads to reduced function of the Act1 adaptor protein, which is essential in IL-17 receptor signal transduction. (5) Activation of IL-36R recruits IRAK proteins and MyD88, which activate the IKK complex and MAPK. The latter activates AP1 in the nucleus, increasing pro-inflammatory gene expression. (6) Upon binding of IL-23 to its receptor complex, Tyk2 and JAK2 activate and phosphorylate STAT3, which dimerizes, enters the nucleus, and promotes the expression of proinflammatory genes and RORγt. JAK inhibitors disrupt this signaling by targeting Tyk2/JAK2. RORγt inhibition leads to decreased Th17 differentiation and response. (7) Sphingosine-1-phosphate signals through its G-protein-coupled receptors (S1P1-5R), activating multiple guanine nucleotide-binding proteins (G proteins) bound to the receptors. Gi activates PLC, cAMP, AKT, Rac, and Ras. The latter leads to downstream signaling via Raf, MEK, and ERK. Gq also activates PLC. PLC leads to PKC activation. G12/13 activates the small GTPase Rho, which binds and activates ROCK/ROK complex. (8) Tapinarof binds to AhR, forming a complex that heterodimerizes with ARNT, leading to downregulation of inflammatory cytokines and upregulation of skin barrier proteins. Created with BioRender.com (accessed on 31 January 2024). Abbreviations: A3AR, A3 adenosine receptor; AhR, aryl hydrocarbon receptor; Akt, Protein kinase B (PKB); ARNT, AhR nuclear translocator; AP1, activator protein 1; ATP, adenosine triphosphate; cAMP, cyclic adenosine monophosphate; CBP, CREB-binding protein; C/EBPs, CCAAT/enhancer binding proteins; CREB, cAMP-response element-binding protein; ERK, extracellular signal-regulated kinase; G12/13, protein G12/13; Gi, protein Gi; Gq, protein Gq; GPCR, G-protein coupled receptor; GTP, guanosine triphosphate; HSP90, heat shock protein 90; IKK, inhibitor of κB (IκB) kinase complex; JAK2, janus kinase 2; JNK, c-JUN N terminal kinase; MAPK, mitogen-activated protein kinase; MEK, mitogen-activated protein kinase kinase; MyD88, innate immune signal transduction adaptor; NF-kB, nuclear factor kappa light-chain enhancer of activated B cells; PKA, protein kinase A; PKC, protein kinase C; PDE, phosphodiesterase; ROR, retinoic acid-related orphan nuclear receptor; RAS, Rat sarcoma virus; Raf, rapidly accelerated fibrosarcoma; Rho, Ras homologous; ROCK/ROK, Rho-associated protein kinase; TAK1, transforming growth factor-beta (TGFβ)-activated kinase 1; TNF, tumor necrosis factor; TNFR, tumor necrosis factor receptor; TRAF, TNF associated factors; TYK2, tyrosine kinase 2; S1P1-5R, sphingosine 1 phosphate receptor 1–5; STAT, signaling transducers and activators of transcription.

This scoping review summarizes the available data on efficacy and safety of novel oral and topical therapies for psoriasis, either in clinical development or under investigation; they include Jak inhibitors, oral inhibitors of TNF, IL-17, and IL-23, retinoic acid-related orphan receptors (ROR)γT inhibitors, sphingosine-1-phosphate receptor 1 (S1PR) inhibitors, A3 adenosine receptor (A3AR) agonists, heat shock protein (HSP)90 inhibitors, Rho-associated protein kinase (ROCK)2 inhibitors, and phosphodiesterase (PDE) 4 inhibiros, among others (Figure 1).

## 2. Methods

English-language medical literature from 1 January 2018 to 20 December 2023 was searched using PubMed and, including the following terms: “psoriasis”, “treatment”, “oral”, “Jak inhibitors”, “target molecules”, “tofacitinib”, “upadacitinib”, “peficitinib”, “solcitinib”, “abrocitinib”, “baricitinib”, “itacitinib adipate”, “brepocitinib”, “ropsacitinib”, “deucravacitinib”, “BMS-986202”, “SAR-20347”, “TAK-279”, “NDI-034858”, “VTX958”, “oral IL-17 inhibitors”, “LY3509754”, “DC-806”, “LEO 153339”, “oral IL-23 inhibitors”, “oral TNF inhibitors”, “ SAR441566”, “JNJ-77242113”, “retinoic acid related orphan receptor inhibitors”, “VTP-43472”, “JTE-451”, “AUR101”, “BI 730357”, “sphingosine-1-phosphate receptor 1 antagonist”, “ponesimod”, “A3 adenosine receptor agonist”, “piclidenoson”, “heat shock protein 90”, “RGRN-305”, “ROCK-2 inhibitor”, “belumosudil”, “Aryl Hydrocarbon Receptor (AhR) Modulators”, “tapinarof”, “topical benvitimod”, “topical roflumilast”, “topical crisaborole”, “topical tofacitinib”, “topical ruxolitinib”, “topical brepocitinib”, “topical RORγ inverse agonist”, “topical IL-2 inhibitors”, and “amygalin analogue”. Only human studies, including clinical trials, reviews, and systematic reviews, were considered.

The process of selecting publications was performed by three independent researchers (EC, LR, and LP), and any disparities were resolved through consensus. We also searched www.clinicaltrials.gov (accessed on 31 January 2024) for clinical trials on psoriasis, as well as the abstracts of the annual AAD and EADV meetings held in 2021, 2022, and 2023. We also performed online Google searches for the international non-proprietary names and investigational drug names thus identified.

## 3. Emerging Oral Treatments for Psoriasis

Increasing knowledge of the pathogenesis of psoriasis has brought to light an extensive list of cell surface receptors and intracellular pathways that may become potential targets for oral treatments. In this section, we will discuss the available efficacy and safety data of novel (either in clinical development or under investigation) oral therapies for psoriasis, including Jak inhibitors, tumor necrosis factor (TNF) inhibitors, IL-17 inhibitors, IL-23 inhibitors, RORγT inhibitors, S1PR inhibitors, A3AR agonists, HSP90 inhibitors, and ROCK2 inhibitors (Table 1).

### 3.1. Jak Inhibitors

The Janus kinase/signal transducer and activator of transcription (Jak/STAT) pathways play an important role in diverse cellular processes [4]. They mediate the intracellular signaling of cytokines in both physiological and pathological conditions, notably IMIDs such as psoriasis [4,5]. 

Jaks are receptor-associated tyrosine kinases that act intracellularly as signal transducers. Four molecules compose the Jak family: Jak1, Jak2, Jak3, and Tyk2. When a circulating cytokine (e.g., IL-13) binds to its cell surface receptor, two Jak molecules (one from each receptor subunit) pair to form a dimer after a conformational change in the receptor. Jak dimers are composed of two different Jaks (heterodimers), except Jak2, which can also pair with itself (homodimer) [6,7]. Jak dimers phosphorylate the activated cytokine receptor, permitting the attachment, phosphorylation, and dimerization of STAT proteins. Seven distinct STAT proteins have been identified, namely STAT1, STAT2, STAT3, STAT4, STAT5a, STAT5b, and STAT6. Following dimerization, STATs move to the nucleus, where they act as transcription factors, inducing the expression of growth factor and proinflammatory cytokine genes [6,7]. 

Because of the significant involvement of the Jak/STAT pathway in the pathogenesis of IMIDs, numerous molecules with therapeutic potential have been developed; because of their small molecular size, they can be employed orally or topically [4]. 

One of the biggest concerns related to Jak inhibitors is their safety, related to their potential interference with the physiological roles of Jak/STAT pathways and the relative non-selectivity of orthosteric Jak inhibitors (blocking the kinase function of the JH1 catalytic domain). Most adverse events (AEs) observed in both clinical trials and real-world clinical settings correspond to mild and moderately severe infections involving the upper respiratory tract, urinary tract, and gastrointestinal system [4,5,8]. Some AEs of Jak inhibitors are considered class-related, mostly because of the rather limited selectivity of the first approved inhibitors of the Jak family. Jak1 inhibition may increase the serum levels of triglycerides, total cholesterol, low-density lipoprotein cholesterol, and high-density lipoprotein cholesterol. Jak2 mediates the action of growth factors involved in erythropoiesis, myelopoiesis, and platelet activation; hence, Jak2 inhibition can lead to anemia, neutropenia, thrombocytopenia, and thromboembolic events. The distribution of Jak3 is restricted to hematopoietic cells, and its function is exclusively linked with signal transduction of the common γc cytokine receptor subunit for interleukins that mediate the activation, function, and proliferation of lymphocytes. Lastly, Tyk2 inhibition interferes with signaling of type I and type III interferons, IL-6, IL-12, and IL-23, and thus can be associated with an increased risk of herpesvirus, staphylococcal, and mycobacterial infections.

Cytokine signaling involves the interaction of two receptor subunits and the formation of Jak dimers. The Jak2/Tyk2 dimer participates in signal transduction of IL-12 and IL-23; both are relevant in the pathogenesis of psoriasis, especially as regards the regulatory role of IL-23 on production of IL-17, the key effector cytokine in psoriasis [6,7,8]. Jak1/Tyk2 is involved in signal transduction of IFN-α and the IL-10 family, which are involved in the immunopathogenesis and epidermal proliferation of psoriasis. Selective targeting of Tyk2 may avoid production of Jak2-related—hematologic and thromboembolic—AEs [9,10]. 

#### 3.1.1. Tofacitinib

Tofacitinib is an oral Jak1 and Jak3 inhibitor approved for the treatment of moderate-to-severe rheumatoid arthritis (2012), psoriatic arthritis (2017) at doses of 5 mg twice daily in adult patients, and ulcerative colitis at doses of 10 mg twice daily in adult patients by the Food and Drug Administration (FDA) of the United States (US) [11]. All these indications were also approved by the European Medicines Agency (EMA). Furthermore, the EMA also approved tofacitinib for the management of ankylosing spondylitis in adults and polyarticular juvenile idiopathic arthritis in children and adolescents aged 2 years and older. The recommended dosages for both conditions involve an initial period of 10 mg twice daily for the initial eight weeks, followed by a maintenance dose of 5 mg twice daily [12].

Tofacitinib has been evaluated for the treatment of moderate to severe psoriasis in phase II and phase III clinical trials. Tofacitinib was compared with placebo in a phase IIb dose-ranging trial (NCT00678210) that included 197 participants who were randomly assigned to receive tofacitinib twice daily (*bis in die* [BID]) at doses of 2 mg, 5 mg, or 15 mg, or placebo [13]. The primary efficacy endpoint was 75% or more improvement in Psoriasis Area and Severity Index (PASI) with respect to baseline (PASI75 response) at 12 weeks. PASI75 response rates in patients treated with the different doses of tofacitinib (25%, 40.8%, and 66.7%, respectively) were significantly higher compared with placebo (2%). In addition, a PASI90 response was achieved by 22% of patients treated with any dose of tofacitinib [13]. 

Tofacitinib 5 mg and 10 mg BID were compared with subcutaneous etanercept 50 mg twice weekly and placebo in adult patients with plaque psoriasis and baseline PASI ≥ 12 in a randomized, placebo-controlled, non-inferiority phase III trial (NCT01241591) [14]. At week 12, PASI75 was achieved by 39.5%, 63.3%, 58.8%, and 5.6% of the tofacitinib 5 mg BID, tofacitinib 10 mg BID, etanercept 50 mg twice weekly, and placebo, respectively. Tofacitinib 5 mg BID was significantly inferior to etanercept (*p* = 0.0009). In addition, Physician Global Assessment (PGA) of psoriasis response was achieved by 47.1%, 68.2%, 66.3%, and 15%, respectively. Again, the tofacitinib 5 mg BID group showed significantly lower (*p* < 0.0001) results than etanercept; nevertheless, no differences were noted between tofacitinib 10 mg BID and etanercept. Regarding a significant improvement in the Dermatology Life Quality Index (DLQI), no differences were seen among tofacitinib (66.3% and 78.2%, respectively) and etanercept groups (74.7%). Similar rates of AEs were observed in both groups. 

Two phase III clinical trials (OPT Pivotal 1 [901 patients] and OPT Pivotal 2 [960 patients]) [15,16] compared with facitinib 5 mg BID, tofacitinib 10 mg BID, and placebo. At 16 weeks, PASI 75 was achieved by 39.2%, 59.2%, and 9%, respectively, in OPT Pivotal 1. Similar results were observed in OPT Pivotal 2: 46%, 59.6%, and 11.4%, respectively. Tofacitinib 10 mg BID was more effective starting at week 16 and showed a higher PASI75 response rate between weeks 16 and 28, compared with tofacitinib 5 mg BID (68.8% vs. 55.6%, respectively). Among patients who achieved PASI75 at week 16, 74.1% of the 5 mg group and 79.4% of the 10 mg group maintained PASI75 at 52 weeks. Most patients maintained the response for 24 months [16]. At week 16, patients on both regimens showed improvement in ungual psoriasis, pruritus, and DLQI, which was sustained at week 52 [17,18]. Most AEs were of mild to moderate intensity, with nasopharyngitis being the most frequently reported. In the OPT Pivotal 1 trial, two patients receiving the 10 mg dose experienced severe infections (appendicitis, pneumonia, and pyelonephritis). Additionally, in OPT Pivotal 2, three patients treated with the 5 mg dose presented with severe infections (pneumonia, herpes zoster, and erysipelas). OPT1 revealed elevated cholesterol and creatine kinase levels in 2.5%, 2.5%, and 0.6% of the tofacitinib 5 mg BID, tofacitinib 10 mg BID, and placebo groups, respectively. OPT2 exhibited similar results: 5.2%, 5.2%, and 2%, respectively. Mild cytopenia, headache, upper respiratory tract infections, urinary tract infections, and diarrhea were the most prevalent side effects observed with tofacitinib at doses of 5 mg and 10 mg [15,16,17,18]. Furthermore, twelve patients across the tofacitinib groups developed reactivation of herpes zoster; analysis of the distribution by race and region of herpes zoster events observed was not reported.

No real-world studies of tofacitinib for the treatment of psoriasis have been conducted following its rejection by the FDA in October 2015 [19], and the application for EMA approval was dropped. The refusal was based on the stipulation that further studies assessing its long-term safety were deemed necessary.

#### 3.1.2. Abrocitinib

Abrocitinib is an oral Jak1 inhibitor. Abrocitinib was approved in 2021 by the EMA and in 2022 by the FDA for the treatment of atopic dermatitis at the recommended initial dose of 200 mg/day in most patients [20]. The safety and tolerability of abrocitinib were demonstrated in a phase I trial (NCT01835197) [21]. Later, abrocitinib was evaluated in a phase II, placebo-controlled trial (NCT02201524), in which 59 patients with moderate to severe psoriasis were enrolled and randomly assigned to receive either placebo or abrocitinib at doses of 200 mg once a day (*quaque die* [QD]), 400 mg QD, or 200 mg BID [22]. At week 4, PASI75 response was achieved by 17%, 50%, and 60% of the patients treated with 200 mg QD, 400 mg QD, and 200 mg BID, respectively, and 17% of patients in the placebo arm. The predominant AEs reported included nausea, headache, neutropenia, and thrombocytopenia. Eight patients discontinued treatment due to AEs, the majority of which were due to cytopenia (one patient in the placebo group, one patient in the 200 mg QD groups, and six patients in the 200 mg BID group). Laboratory abnormalities, including low neutrophil, reticulocyte, and platelet counts, exhibited a higher frequency in the 200 mg BID group as opposed to the other groups. No serious infections or bleeding events related to neutropenia or thrombocytopenia, respectively, were reported. Abrocitinib has eventually been approved for the treatment of moderate to severe atopic dermatitis in adults who are candidates for systemic therapy, and there is no currently active clinical trial assessing the efficacy of abrocitinib as a therapeutic intervention for psoriasis.

#### 3.1.3. Baricitinib

Baricitinib is an orally administered Jak1 and Jak2 inhibitor. Baricitinib was approved by the FDA and EMA for the treatment of rheumatoid arthritis and severe alopecia areata. Also, the EMA approved baricitinib for the treatment of atopic dermatitis and active juvenile idiopathic arthritis in children aged 2 or older at a recommended dose of 4 mg/day in most patients [23,24].

The tolerability and safety of baricitinib were evaluated in two phase I clinical trials, in which no serious AEs were reported [25]. A phase IIb, randomized, double-blind, placebo-controlled, dose-ranging clinical trial (NCT01490632) evaluated the efficacy of baricitinib for the treatment of moderate to severe psoriasis [26]. A total of 271 patients were included. At 12 weeks, PASI75 response rates were 42.9% (8 mg QD) and 54.1% (10 mg QD), significantly higher than those of patients receiving placebo (16.1%). Infections, particularly nasopharyngitis, were the most commonly reported AEs [26]: nasopharyngitis was reported in 11.8%, 3.1%, 2.8%, 9.4%, and 8.7% of the treatment groups: placebo, baricitinib 2 mg, 4 mg, 8 mg, and 10 mg, respectively. Treatment-emergent AEs were similar in the placebo, 2 mg, and 4 mg dose groups (44.1%, 50.0%, and 47.2%, respectively), but were higher in the 8 mg and 10 mg dose groups (57.8% and 63.8%, respectively). There is currently no ongoing clinical trial evaluating baricitinib for the treatment of psoriasis. 

#### 3.1.4. Peficitinib

Peficitinib is an orally administered Jak inhibitor with moderate selectivity for Jak3 and Jak1 over Jak2 and Tyk2 [27]. A phase IIa (NCT01096862) randomized, placebo-controlled, double-blind trial evaluated the safety and efficacy of peficitinib in patients with moderate to severe psoriasis [28]. A total of 124 participants were enrolled and randomly allocated across five groups, with four receiving peficitinib 10 mg, 25 mg, 60 mg, and 100 mg BID, and one receiving 50 mg once daily (QD) as active treatment. Patients within each group were randomly allocated to peficitinib or placebo in a 3:1 ratio (namely, eighteen receiving peficitinib and six receiving placebo). After 6 weeks, all peficitinib groups exhibited a higher PASI reduction compared with placebo and a dose-dependent response, with the 100 mg BID group showing the greatest PASI reduction [28]. 

PASI75 response was achieved by 58.8% of individuals in the 100 mg BID group and 14.3% in the 25 mg BID group (representing the lowest response among the peficitinib groups); the response rates were in all cases significantly higher than those in the placebo group (3.4%). Peficitinib was well tolerated, with all AEs classified as mild or moderate. The most reported AEs included nasopharyngitis (10% in the placebo group and 18% in the 100 mg BID), diarrhea (7% in the placebo group and 6% in the 100 mg BID), acne (0% vs. 18%, respectively), back pain (0% vs. 12%, respectively), and contact dermatitis (7% vs. 0%, respectively) [28].

There is currently no ongoing clinical trial evaluating peficitinib as a treatment for psoriasis.

#### 3.1.5. Solcitinib (GSK2586184)

Solcitinib is an orally administered Jak inhibitor with selectivity for Jak1 over Jak2 [29]. The efficacy and safety of solcitinib in patients with moderate to severe psoriasis were assessed in a phase IIa randomized placebo-controlled trial that enrolled a total of 68 participants (NCT01782664). Patients were randomly allocated to four cohorts, receiving either 100 mg, 200 mg, or 400 mg of solcitinib BID or a placebo. At week 12, PASI75 response was achieved by 0% of individuals in the placebo group, contrasting with 13%, 25%, and 57% of those in the 100 mg, 200 mg, and 400 mg solcitinib BID groups, respectively [29]. The incidence of AEs was similar among the treatment groups, with no discernible correlation between doses and the frequency of AEs. Headache, nasopharyngitis, nausea, diarrhea, fatigue, and upper abdominal pain were the most reported AEs [29].

There is currently no ongoing clinical trial evaluating solcitinib as a treatment for psoriasis.

#### 3.1.6. Itacitinib Adipate

Itacitinib adipate is an orally administered Jak1 inhibitor. The efficacy and safety of itacitinib adipate in psoriasis were evaluated in a randomized, double-blind, placebo-controlled, phase II trial (NCT01634087) that included 50 patients [30]. PASI75 response was evaluated at 4 weeks, with a 0% response rate in the placebo group and 11.1%, 22.2%, and 27.7% in patients treated with Itacitinib adipate at doses of 100 mg QD, 200 mg QD, 200 mg BID, and 600 mg QD, respectively. However, only in the latter group was the difference significant compared with placebo. No relevant AEs were documented, the most frequent being nasopharyngitis (8.3% in placebo vs. 18.3% in itacitinib groups), elevated serum levels of aspartate aminotransferase (0 vs. 5.3%), headache (0 vs. 5.3%), and hypertriglyceridemia (0 vs. 5.3%). [30]. At present, there is no active clinical trial investigating the efficacy of itacitinib adipate as a therapeutic approach for psoriasis.

#### 3.1.7. Brepocitinib

Brepocitinib (formerly PF-06700841) is an oral Tyk2 and Jak1 inhibitor that binds to the active sites of the Tyk2 catalytic domain [31]. A phase I trial confirmed the safety and tolerability of brepocitinib (NCT02310750) [32]. In a phase IIa trial (NCT02969018), 212 patients were enrolled and treated for four weeks with brepocitinib 30 mg QD, brepocitinib 60 mg QD, or placebo. Later, patients were randomly allocated and treated for 8 weeks with placebo, brepocitinib 10 mg QD, 30 mg QD, or 100 mg once weekly [33]. At 12 weeks, PASI75 was evaluated, which was achieved by: 60% of the 60 mg QD followed by 30 mg QD group, 24.1% of the 60 mg QD followed by 10 mg QD group, 57.7% of the 60 mg QD followed by 100 mg once weekly group, 24% of the 60 mg QD followed by placebo group, 86.2% of the 30 mg QD group, 24% of the 30 mg QD followed by 10 mg QD group, 36.7% of the 30 mg QD followed by 100 mg once weekly group, and 13% of the placebo group. Commonly reported AEs without differences among groups included nasopharyngitis, upper respiratory tract infection, and headache. Notably, there were no instances of herpes zoster infections reported, indicating a favorable tolerability profile for the treatment.

In June 2022, Pfizer granted Priovant global developmental rights for both oral and topical formulations, as well as commercial rights in the U.S. and Japan for brepocitinib [34]. The progression of oral brepocitinib has been halted for most of its potential applications, spanning psoriasis, psoriatic arthritis, vitiligo, ulcerative colitis, hidradenitis suppurativa, and Crohn’s disease.

#### 3.1.8. Ropsacitinib

Ropsacitinib (formerly PF-06826647) is an oral inhibitor of Tyk2 and Jak2 that binds to the active site in the catalytic domain (JH1) of each kinase, but with higher selectivity for Tyk2 [31]. The efficacy and safety of ropsacitinib in moderate to severe psoriasis were evaluated in two phase I trials (NCT02310750, NCT03210961) [35,36]. Subsequently, a randomized, double-blind, placebo-controlled phase IIb trial (NCT03895372) included 178 patients who were randomly allocated to receive placebo or ropsacitinib at doses of 50 mg, 100 mg, 200 mg, or 400 mg QD. [37]. The PASI75 response was assessed at week 16, with the 50 mg, 100 mg, 200 mg, and 400 mg QD cohorts achieving 18.2%, 9.5%, 46.7%, and 73.2%, respectively. Results from the 200 mg and 400 mg QD groups were significantly higher than those observed with placebo (14.3%). Nasopharyngitis (4.5% in the placebo group vs. 27.7% in the 400 mg QD group) and increased blood pressure were the most frequently reported AEs (4.35% in the placebo group vs. 11.63% in the 400 mg QD group). [37]. At present, there is no active clinical trial involving ropsacitinib, which has also been licensed by Pfizer to Priovant [34]. 

#### 3.1.9. Deucravacitinib

Deucravacitinib is an oral allosteric Tyk2 inhibitor that binds to the pseudokinase or regulatory (JH2) domain, causing a conformational change that prevents the catalytic activity of the kinase (JH1) domain [38,39,40,41]. Consequently, deucravacitinib displays a great selectivity for Tyk2 over Jak1/3 (1000-fold) and Jak2 (2000-fold) [39,40]. 

Deucravacitinib was evaluated for the treatment of moderate to severe psoriasis in a randomized, placebo-controlled, phase IIa trial [42]. A total of 267 patients were included and randomly assigned to receive placebo or deucravacitinib with four dosing arms: 3 mg BID, 6 mg BID, and 12 mg QD. PASI75 response was evaluated at week 12 and achieved by 6.7% (placebo), 68.9% (3 mg BID), 66.7% (6 mg BID), and 75% (12 mg QD) of patients. The reported AEs were considered mild in all cases, and the most frequently reported were nasopharyngitis, headaches, diarrhea, nausea, and upper respiratory tract infections. No alterations in serum lipid levels or hematologic abnormalities were noted, as would correspond to the high selectivity of deucravacitinib for Tyk2 with negligible Jak1 or Jak2 interaction [42,43]. 

Two phase III trials compared deucravacitinib to placebo and apremilast for the treatment of moderate to severe psoriasis [44]. POETyk PSO-1 (NCT03624127) was a double-blind, 52-week trial with 666 patients who were randomly assigned in a 2:1:1 pattern to deucravacitinib 6 mg QD (n = 332), placebo (n = 166), or apremilast 30 mg BID (n = 168). At week 16, deucravacitinib showed significantly higher PASI75 response rates (58.4%) than placebo (12.7%) and apremilast (35.1%). Moreover, the efficacy of deucravacitinib improved beyond week 16 and was maintained through week 52 (68.3%). At week 24, PASI75 was achieved by 69.3% of the patients in the deucravacitinib group, compared with only 38.1% of the patients in the apremilast group. The most frequent AEs related to deucravacitinib were nasopharyngitis and upper respiratory tract infections. Similar rates were noted among the three groups: placebo, deucravacitinib, and apremilast: 4.2%, 6.3%, and 8.3%, respectively, for nasopharyngitis and 3.6%, 6.3%, and 1.8%, respectively, for upper respiratory tract infections. POETyk PSO-2 (NCT03611751) [45] was a 52-week trial that included 1020 participants randomized into three groups: deucravacitinib 6 mg QD (511), placebo (255), and apremilast 30 mg BID (254). Once more, deucravacitinib demonstrated superiority with a higher PASI75 response rate (53%) than placebo (9.4%) and apremilast (39.8%) at week 16. The efficacy persisted through week 52 with ongoing deucravacitinib administration. Nasopharyngitis was the most frequently reported adverse event, and no significant laboratory abnormalities were observed [45]. 

Armstrong et al. have recently published a systematic review and network meta-analysis in which deucravacitinib was indirectly compared with other systemic biologic and nonbiologic therapies [46]. Deucravacitinib PASI75 response at short-term (10–16 weeks) was 54.1% (46.5–61.6). These results were similar to the results observed with the first-generation of biologics: etanercept 39.7% (31.6–48.3) and infliximab 79.0% (74.0–83.5). At long-term follow-up (44–60 weeks), deucravacitinib PASI75 was 65.9% (58.0–73.4), also close to the PASI75 achieved by first-generation biologics adalimumab 62.8% (55.3–69.6) and ustekinumab 68.0% (64.6–71.5). Furthermore, a matching-adjusted indirect comparison (MAIC) of the long-term efficacy of deucravacitinib versus adalimumab for patients with moderate-to-severe plaque psoriasis was recently published [47]. This MAIC concluded that patients treated with deucravacitinib showed a higher long-term response rate at 2 years than with adalimumab. Adalimumab response rates declined by year two, whereas deucravacitinib response rates remained stable. 

Several phase III and 4 trials are currently assessing the efficacy and safety of deucravacitinib in the treatment of moderate to severe psoriasis (NCT04036435), scalp psoriasis (NCT05478499), nail psoriasis (NCT05124080), and in pediatric patients with moderate to severe psoriasis (NCT04772079). Additionally, an upcoming phase IV observational post-marketing surveillance study will focus on AEs in patients with psoriasis in Japan (NCT05633264). Finally, a trial examining adherence in patients with psoriasis is expected to commence recruitment soon (NCT05570955).

Deucravacitinib 6 mg daily was approved by the FDA (2022) and EMA (2023) for the treatment of moderate to severe psoriasis [48,49,50]. In 2022, deucravacitinib was approved in Japan for the treatment of plaque psoriasis, generalized pustular psoriasis, and erythrodermic psoriasis. 

#### 3.1.10. BMS-986202

BMS-986202 is an oral Tyk2 inhibitor created by structural and molecular modifications applied to deucravacitinib [51] and shares its mechanism of action. Encouraging results have been observed in preclinical studies, but no clinical studies assessing its efficacy have been conducted yet [51]. A phase I trial testing its safety, tolerability, pharmacokinetics, and pharmacodynamics is already complete, but results have not been posted. 

#### 3.1.11. SAR-20347

SAR-20347 is an orally administered Tyk2 and Jak1 inhibitor that has been shown to be effective in attenuating pathologic alterations in the imiquimod murine model of psoriasis [52]. No trials testing its efficacy and safety have been performed yet. 

#### 3.1.12. Zasocitinib (TAK-279)

Zasocitinib (TAK-279) is an oral allosteric Tyk2 inhibitor developed by Nimbus Therapeutics (NDI-034858) and acquired by Takeda in 2022 [53]. A phase II, randomized, double-blind, placebo-controlled trial (NCT04999839) evaluated its efficacy, safety, and tolerability [54]. A total of 259 patients were enrolled and distributed among five groups: zasocitinib at doses of 2 mg, 5 mg, 15 mg, and 30 mg QD, and placebo. By week 12, PASI75 response rates for all zasocitinib groups (44%, 68%, and 67% for 5 mg, 15 mg, and 30 mg, respectively) were significantly higher compared with the placebo group (6%), except those of the 2 mg QD group [54].

Currently, two phase III trials that will evaluate zasocitinib efficacy, safety, and tolerability (NCT06088043 and NCT06108544) are still recruiting patients. NCT06108544 will compare zasocitinib to placebo and apremilast. 

#### 3.1.13. VTX958

VTX958 is an orally administered selective allosteric Tyk2 inhibitor developed by Ventyx Bioscience. Results of a randomized, multicenter, double-blind, placebo-controlled phase II trial in moderate to severe psoriasis (NCT05655299) with four oral doses (50 mg BID, 300 mg QD, 225 mg BID, and 300 mg BID) have been released. Even though PASI75 response rates with the two higher doses were significantly superior to placebo at week 16, the results have not met the sponsor’s expectations, and further development on psoriasis and psoriatic arthritis has been terminated [55]. 

### 3.2. Oral PDE4 Inhibitors

Apremilast was the first PDE4 inhibitor approved for the treatment of psoriasis in 2014 [56]. Roflumilast, another PDE4 inhibitor initially approved for chronic obstructive pulmonary disease with available generics, has also demonstrated its efficacy in the treatment of psoriasis in an investigator-initiated clinical trial [57], and new oral PDE4 inhibitors are being developed, such as orismilast [58], Hemay005 (mufemilast), and ME3183 [59]. 

Orismilast is a potent oral PDE4 inhibitor with enhanced selectivity for the PDE4B and PDE4D subtypes. A randomized, double-blind, placebo-controlled phase IIa trial involved 36 patients with moderate to severe psoriasis, randomly assigned to receive either placebo or orismilast 30 mg BID [58]. By week 16, 44.4% of patients receiving orismilast achieved PASI75, versus 5.6% in the placebo group. The predominant AE reported in the orismilast group included nausea and diarrhea [58]. Subsequently, a phase IIb randomized clinical trial enrolled 202 patients to assess the efficacy and safety of orismilast in individuals with moderate-to-severe plaque psoriasis (NCT05190419). Patients were randomly assigned to receive placebo or orismilast at doses of 20 mg, 30 mg, or 40 mg BID. The results of the trial have been published on the www.clinicaltrials.org (accessed on 31 January 2024) website, showing superiority of all doses vs. placebo regarding percent change from baseline in PASI score at week 16, but inconsistencies have been detected by the Food and Drug Administration quality control review.

Hemay005 (mufemilast) is an oral PDE4 inhibitor being developed for psoriasis treatment. A randomized, placebo-controlled phase II trial evaluated Hemay005’s safety and efficacy in patients with moderate to severe psoriasis (NCT04102241). A total of 216 patients were enrolled and divided into 4 groups: placebo and Hemay005 (15 mg, 30 mg, and 60 mg). However, results have not been posted yet. In addition, a randomized, multicenter, double-blind, placebo-controlled phase III trial will also evaluate its efficacy and safety in patients with moderate to severe psoriasis (NCT04839328). This trial will divide patients into two groups: placebo and Hemay005 60 mg BID. Currently, the trial is still recruiting patients. 

ME3183 is an oral PDE4 inhibitor under development for the treatment of psoriasis, atopic dermatitis, and other inflammatory diseases [59,60]. Two double-blind, placebo-controlled, single-ascending doses and multiple-ascending doses phase I studies (ME3183-1 and ME3183-2) evaluated its safety, tolerability, and pharmacokinetics. A total of 126 healthy patients were included. ME3183 demonstrated safety and tolerability up to a dosage of 25 mg (single dose) and up to 10 mg twice daily. Commonly reported treatment-emergent AE encompassed diarrhea and headache, aligning with established patterns associated with approved PDE4 inhibitors, thereby presenting no unprecedented safety considerations. A multicenter, randomized, double-blind, placebo-controlled, parallel group, phase IIa study, in which 136 patients were enrolled, evaluated ME3183 safety and efficacy (NCT05268016). Patients were divided into placebo and ME3183 (5 mg BID), 7.5 mg BID, 10 mg QD, and 15 mg QD [61]. PASI75 was evaluated after 16 weeks of treatment (primary endpoint). A significantly greater proportion of patients treated with ME3183 (5 mg BID, 7.5 mg BID, and 15 mg QD) achieved PASI75 compared with placebo (58.3%, 61.5%, and 52.0% vs. 14.8%, respectively). No differences were noted between ME3183 10 mg QD and placebo. Furthermore, a greater proportion of patients in the ME3183 groups versus placebo achieved PASI90 and PASI100. ME3183 was well tolerated, and the most frequent treatment-emergent AE reported were diarrhea, nausea, and headache.

#### 3.2.1. Oral TNF Inhibitors

Since TNF participates in the pathogenesis of several IMIDs, research has focused on the development of oral agents inhibiting this key factor of the inflammatory pathways [62]. Small molecules that promote the stabilization of an asymmetrical configuration of the soluble TNF trimer may lead to attenuation of TNF signaling, consequently inhibiting TNF function [63,64].

Sanofi is currently working on SAR441566, an oral TNF inhibitor [65,66]. A randomized, double-blind, placebo-controlled phase I trial with 38 participants evaluated the efficacy and safety of SAR441566 in patients with moderate to severe psoriasis (NCT05453942). The trial is complete, but results have not been posted yet. 

#### 3.2.2. Oral IL-17 Inhibitors

Biologic drugs have made a huge impact in the management of psoriasis as well as in patients’ quality of life. Therefore, research has focused on oral molecules directed at the same targets [67]. The intricate modulation of extensive protein-protein interactions through small molecules poses a complex challenge, but numerous research groups are dedicated to pursuing this objective [67,68].

At least three different molecules have been developed independently by Lilly, Leo Pharma, and DICE Therapeutics. In 2019, two phase I clinical trials with LY3509754 (Lilly) in psoriasis (NCT04152382, NCT04586920) were prematurely concluded due to safety issues associated with liver function [69]. The safety and tolerability of LEO 153339 (Leo Pharma) [67,68] were evaluated in a randomized, double-blind, phase I trial in which 108 participants with moderate to severe psoriasis were included (NCT04883333). The study was completed in July 2023, but results have not been posted yet. 

The safety and pharmacokinetic properties of DC-806, an oral IL-17 inhibitor developed by DICE Therapeutics, were evaluated in a randomized, double-blind, placebo-controlled phase I trial in healthy volunteers [70]. The study encompassed three sequential parts: phase Ia involved a single ascending dose (n = 40), phase Ib comprised multiple ascending doses (n = 32), and phase Ic focused on proof-of-concept in psoriasis patients (n = 32). In the latter phase, patients were stratified into three groups: the high-dose group (comprising eight patients who received 800 mg BID), the low-dose group (comprising thirteen patients who received 200 mg BID), and the placebo group, which included eleven patients. PASI reduction at 4 weeks of treatment was significantly higher (*p* = 0.0008) in the high dose group (43.7%) compared with the placebo group (13.3%). Furthermore, both regimens of DC-806 showed a dose-dependent inhibition of IL-17 [70]. DICE Therapeutics is reportedly developing a fast follower of DC-806 and DC-853 with improved potency and metabolic stability, and Lilly has recently completed the acquisition of DICE Therapeutics with its DELSCAPE DNA-encoded library-based platform to discover small molecules targeting protein-protein interactions [71]. 

### 3.3. Oral IL-23 Inhibitors

Protagonist Therapeutics, in collaboration with Janssen Biotech, Inc., is developing JNJ-77242113, an oral IL-23 receptor antagonist peptide [72]. The results of an initial phase I trial (NCT05062200) were promising, and two phase II trials were started [73]. SUMMIT (NCT05357755) is a multicenter, randomized, double-blind, placebo-controlled phase IIa trial on 90 patients with moderate to severe psoriasis comparing a delayed-release tablet of JNJ-77242113 with placebo in adults with moderate-to-severe plaque psoriasis; its results have not been posted yet. FRONTIER 1 (NCT05223868), a multicenter, randomized, placebo-controlled, dose-ranging phase IIb trial that included 255 participants, evaluated the efficacy and safety of JNJ-77242113 in patients with moderate-to-severe plaque psoriasis. At week 16, PASI75 response rates (primary endpoint) were 37.2% at 25 mg QD, 51.2% at 25 mg BID, 58.1% at 50 mg QD, 65.1% at 100 mg QD, 78.6% at 100 mg BID, and placebo 9.3% (nominal *p* ≤ 0.002 for all comparisons); the corresponding PASI90 response rates were 25.6%, 26.8%, 51.2%, 46.5%, 59.5%, and 2.3%, respectively, and PASI100 response rates were 11.6%, 9.8%, 25.6%, 23.3%, 40.5%, and 0%, respectively [74]. No differences in AEs were noted among the placebo and the five treatment groups [73]. If confirmed in phase III trials, these levels of response would set a new standard of efficacy for oral treatments of moderate to severe psoriasis. Currently, two additional trials of JNJ-77242113 are in progress: a phase I trial involving a single dose to assess the pharmacokinetics, safety, and tolerability (NCT05703841), and FRONTIER 2 (NCT05364554), a phase II trial intended to evaluate the efficacy and safety of JNJ-77242113 at week 36 [73].

### 3.4. RORγT Inhibitors

The retinoic acid-related orphan receptors (ROR, including RORα, RORβ, and RORγ) function as transcription factors upon binding with their ligands [75,76]. The union of IL-23 to its receptor activates STAT3 and induces the expression of RORγT, an isoform of RORγ that promotes a Th17 response with transcription of IL-17A, IL-17F, IL-22, and IL-23R [77]. Inhibiting Th17 differentiation reverts this commitment and promotes the expansion of functional regulatory T cells that release anti-inflammatory cytokines, such as IL-10, thereby dampening the immune response [78]. Consequently, RORγ has been identified as a potential target for modulating the Th17 response.

Several RORγT inverse agonists have been tested on clinical trials for the treatment of moderate to severe psoriasis, with modest success to date: VTP-43742, JTE-451, AUR101, ABBV-157, IMU-935, BMS-986251, AZD0284, SAR441169, ABBV-553, and BI 730357 [75,79,80,81].

VTP-43742 was associated with a 30% reduction from baseline PASI after 4 weeks of treatment in a phase I clinical trial (NCT03724292), but the study had to be discontinued due to liver toxicity. The phase I/II studies evaluating ABBV-553 (NCT03145948), BMS-986251 (NCT03329885), and ABBV-157 (cedirogant) (NCT05044234) were also terminated due to undisclosed safety reasons, while phase I trials on AZD0284 (NCT03310320), SAR441169, and IMU-935 were terminated due to limited efficacy.

A randomized, placebo-controlled phase II trial evaluated JTE-451 (NCT03832738). A total of 152 patients were enrolled and divided into three groups (200 mg BID, 400 mg BID, or placebo). At 16 weeks, PASI75 (primary endpoint) was achieved by 11.8%, 22%, and 7.8%, respectively. No phase III trials have been started to date.

Regarding AUR101, two phase II trials (NCT04207801 and NCT04855721) were performed. NCT04207801 was a double-blind, placebo-controlled, randomized, multicentric phase II trial involving 90 participants who were allocated to three treatment arms: 400 mg BID, 800 mg BID, or placebo. By week 12, the PASI75 response was attained by 60%, 63.3%, and 26.7% of individuals in the respective treatment groups. INDUS-3 (NCT04855721) was a randomized, double-blind, placebo-controlled, phase IIb clinical trial that evaluated three doses of AUR101 (200 mg BID, 400 mg QD, and 400 mg BID) with disappointing results; only the 400 mg BID group was superior to placebo regarding the primary endpoint (PASI75), and further development of AUR101 by Aurigene Oncology was halted [82].

Two phase II trials investigating BI 730357 in psoriasis (NCT03635099 and NCT03835481) have been completed. NCT03635099 was a randomized, double-blind, placebo-controlled phase II trial that assessed the safety, tolerability, and efficacy of BI 730357 in individuals with moderate to severe psoriasis [83]. A total of 274 participants were enrolled and distributed across eight treatment arms, considering the fasting status upon administration: placebo (fasted), 25 mg (fasted), 50 mg (fasted), 100 mg (fasted), 200 mg (fasted), placebo (fed), 400 mg (fed), and 200 mg BID (fed). At week 12, the PASI75 response was achieved by 0%, 5%, 7.7%, 10.3%, 30%, 0%, 25.6%, and 23.8% of patients in the respective groups. An upper respiratory tract infection was reported in 21 patients [83]. NCT03835481 was a long-term extension trial in patients with moderate to severe psoriasis that had completed the preceding trial (NCT03635099); it enrolled 165 patients, some of whom were up-dosed. The primary outcome was treatment-induced emergent AEs, which were reported in 60.9% of the patients in the higher dose regimen (400 mg), with a 37.5% PASI75 response rate. The study was terminated due to the sponsor’s decision, and no further trials have been conducted to date.

Other molecules under development, like VTP-45489, have not yet been evaluated in clinical trials.

### 3.5. Sphingosine-1-Phosphate Receptor 1 Antagonist

Sphingosine-1-phosphate (S1P) is a bioactive lipid that binds five G-protein-coupled receptors and governs essential cellular functions, including proliferation, survival, migration, and adhesion [84,85]. The S1P_1_ receptor (S1P_1_R) is notably expressed in the skin, lymphoid tissue, and cardiovascular system [86,87]. Ponesimod, an oral, selective modulator of S1P_1_R, has demonstrated efficacy in diminishing the populations of circulating T and B cells, particularly CD4+ cells, in healthy human subjects [88,89] and has been tested in two phase II trials (NCT01208090 and NCT00852670) for treatment of moderate to severe psoriasis. NCT01208090 was a randomized, multicenter, double-blind, placebo-controlled phase II trial with 326 patients who were allocated to three treatment groups: 20 mg QD, 40 mg BID, or placebo. PASI75 at 16 weeks of treatment (primary endpoint) was achieved by 46%, 48.1%, and 13.4% of the patients in each group, respectively (*p* < 0.0001 versus placebo for both active treatment arms) [90]. Subsequently, patients were re-randomized during the maintenance period to receive: 20 mg, 40 mg, or placebo. By the 28th week of the follow-up period, 71.4% of patients maintaining the 20 mg dosage and 77.4% of those continuing with 40 mg achieved a PASI75 response. Conversely, individuals re-randomized to the placebo from the 20 mg and 40 mg groups exhibited a swift decline in effectiveness, with PASI75 attainment recorded at 42.2% and 40.4% for patients initially treated with ponesimod 20 mg and 40 mg, respectively. Dyspnea, liver enzyme abnormalities, and dizziness were the most frequently reported AEs [90]. Results from NCT00852670 have not been published yet. Currently, there is no ongoing clinical trial for ponesimod.

### 3.6. A3 Adenosine Receptor Agonist

A3 adenosine receptor (A3AR) is a Gi protein-coupled receptor that can be found on the cell surface and is overexpressed in inflammatory skin conditions as well as in peripheral blood mononuclear cells [91].

Piclidenoson (formerly named CF101) is an orally administered, water-insoluble agonist of the A3 adenosine receptor (A3AR) [91,92]. Upon activation of the A3AR, there is a downregulation of the NF-κB signaling pathway, resulting in a decrease in the expression of inflammatory cytokines such as TNF, IL-12, IL-17, and IL-23. Consequently, piclidenoson induces an anti-inflammatory effect and inhibits keratinocyte proliferation [76,93]. In fact, activation of A3AR is the predominant mechanism of action of methotrexate in IMIDs [94]. Piclidenoson has already been evaluated in phase II and phase III trials with satisfactory results [95].

Firstly, in a multicentric, double-blinded, placebo-controlled phase II trial (NCT00428974), 75 patients with moderate to severe psoriasis were included. Patients were randomized to receive a placebo or piclidenoson 1, 2, or 4 mg BID. At 12 weeks of treatment, the 2 mg BID group showed a statistically significant reduction in PASI score from baseline compared with the placebo. PASI50 was achieved by 35.3% of the patients treated with the 2 mg BID regimen. The 1 mg dose exhibited no therapeutic effect, and the 4 mg group exhibited less improvement compared with the 2 mg group.

Subsequently, a randomized, double-blind, placebo-controlled, phase II/III study (NCT01265667) was carried out [95]. Patients were randomly allocated to parallel dosing groups receiving CF101 2 mg or corresponding placebo tablets BID. Despite not achieving the primary study endpoint (statistically significant improvement in PASI75 response rate compared with placebo at week 12), subsequent data analysis uncovered statistically significant cumulative and linear improvement from weeks 16 to 32. By week 32, 33%, 25%, and 11% of patients treated with CF101 2 mg BID achieved PASI75, PASI90, and PASI100 responses, respectively. During the follow-up period, the treatment was also well-tolerated. In addition, an indirect comparison was conducted between the data from the piclidenoson phase II/III trial and the data from the apremilast phase III trials [95]. The efficacy of apremilast plateaued at week 16, with 30% of patients attaining PASI75 response, while piclidenoson presented a response rate of 35.3% in terms of PASI75 at week 32, showing no visible plateau.

Recently, results from COMFORT (NCT03168256), a multicenter, randomized, phase III placebo- and active (apremilast)-controlled trial, have been published. A total of 529 patients were included and randomized into four treatment groups: placebo, CF101 2 mg BID, CF101 3 mg BID, and apremilast 30 mg BID. PASI75 was evaluated at week 16, and results are posted on the www.clinicaltrials.org (accessed on 31 January 2024) website. However, inconsistencies have been detected by the Food and Drug Administration quality control review. 

### 3.7. Heat Shock Protein 90

Heat shock protein 90 (HSP90), one of the most ubiquitous chaperone proteins, participates in folding, stabilizing, and activating substrate proteins, such as transcriptional factors and intracellular signaling molecules that mediate inflammation [76]. Increased expression of Heat Shock Proteins (HSP) across distinct layers of the skin is believed to play a role in the pathogenesis of psoriasis; interestingly, the number of psoriasis flares over the course of one year appears to correlate with an increase in HSP90 expression [96,97]. 

RGRN-305 (CUDC-305) is a HSP90 inhibitor with promising results in a xenograft mouse model of psoriasis [98]. In vitro studies demonstrated a reduction in the expression of genes coding for inflammatory cytokines such as TNF or IL-23 [99]. In an open-label, single-arm, dose-selection, single-center proof-of-concept phase Ib trial (NCT03675542), 11 patients with psoriasis were treated with RGRN-305 250 mg or 500 mg QD [100]. After 12 weeks of treatment, six out of eleven patients showed ≥50% improvement (range 71–94%) with respect to baseline PASI, without a clear dose effect. Although four of the seven patients treated with the 500 mg dose developed an exanthematous reaction, no serious AEs were reported. Furthermore, a skin transcriptome analysis disclosed a prompt and maintained decrease of relevant inflammatory transcripts induced by TNF and IL-17, such as IL36G and CXCL8.

Inhibition of HSP90, even as a topical treatment option, could emerge as an innovative therapeutic strategy, applicable not only to psoriasis but also to various other immune-mediated skin disorders [101]. 

### 3.8. ROCK-2 Inhibitor

The Rho-associated protein kinases (ROCK) 1 and 2 are the downstream mediators of Rho proteins, which conform to a GTP-binding protein family [102]. These kinases play a pivotal role in various cellular processes, encompassing cell migration, adhesion, proliferation, and apoptosis [102]. The anti-inflammatory effect of ROCK2 inhibitors is mediated by a downregulation of the T cell response [12].

Belumosudil (KD025, Rezurock^®^) is a ROCK inhibitor formulated by Kadmon Pharmaceuticals [103]. It became the first selective ROCK-2 inhibitor with a 100-fold specificity for ROCK-2 over ROCK-1. In addition, compared with dual ROCK inhibitors, belumosudil possesses an improved safety profile [102]. KD025 has been tested in multiple phase I trials with healthy volunteers, with good tolerance and no serious AEs, including cardiovascular side effects [104,105]. Subsequently, belumosudil has been tested in multiple phase II clinical trials for the treatment of chronic graft-versus-host disease, idiopathic pulmonary fibrosis, systemic sclerosis, and psoriasis (NCT02317627). The latter was an open-label, phase II trial in which 38 patients were included and randomized into three groups: 200 mg belumosudil BID, 400 mg belumosudil QD, and 400 mg belumosudil BID. After 12 weeks of treatment, a PASI50 response was achieved by 71%, 42%, and 29% of patients in each treatment arm, respectively, suggesting a higher benefit with the lower dosage strategy. However, the PASI75 response was achieved only by 14.2%, 16.7%, and 14.2% of patients, respectively. These results are apparently inferior to those that can be achieved with methotrexate, apremilast, or Jak inhibitors. Diarrhea was the most frequently reported adverse event. Two additional phase II double-blind, placebo-controlled studies have recently been conducted (NCT02852967, NCT02106195). NCT02852967 included 110 patients, which were randomized to receive placebo, belumosudil 200 mg QD, 200 mg BID, 400 mg QD, or 600 mg/day. PASI75 response was evaluated after 16 weeks of treatment (primary endpoint) and was achieved by 55.6%, 60.9%, 68.2%, 81.0%, and 61.5% of patients in the respective treatment arms. Despite the numerically high response rate in the 400 mg QD group (PASI75 81.0%), no significant superiority to placebo could be demonstrated. Apart from occasional serious AEs such as natural death (n = 1), pneumonia (n = 1), hypercholesterolemia (n = 1), thalamic infarction (n = 1), and chronic obstructive pulmonary disease (n = 1), the treatment was well tolerated, and the most frequently reported AEs were headache, nausea, increased serum levels of liver enzymes, and upper respiratory tract infections. NCT02106195 included eight patients and was intended to determine the safety and tolerability of belumosudil. Two patients had to discontinue the treatment due to AEs. 

## 4. Current and Emerging Topical Treatments for Psoriasis

Corticosteroids and analogues of vitamin D persist as the primary topical therapeutic option for patients with mild-to-moderate psoriasis [106,107]. Topical corticosteroids induce anti-inflammatory, anti-proliferative, and localized vasoconstrictive effects by suppressing genes that encode proinflammatory cytokines, while vitamin D analogues (such as calcipotriol and tacalcitol) decrease keratinocyte proliferation and regulate both humoral and cellular immune responses through the inhibition of proinflammatory cytokines from T-cells [108,109]. The combination of these agents has shown higher efficacy compared with their individual applications in monotherapy [110,111]. In addition, the concurrent use of corticosteroids mitigates the irritative consequences associated with vitamin D analogues, while the potential for atrophy induced by corticosteroids is limited by the action of vitamin D analogues [110,111].

Other topical options that are currently available for psoriasis include topical retinoids, like tazarotene, and topical calcineurin inhibitors, such as pimecrolimus and tacrolimus. The former works by inhibiting keratinocyte proliferation, although its effect is limited by local irritation. New formulations based on lower doses of tazarotene and in combination with corticosteroids have shown increased efficacy and tolerance [112,113]. Topical calcineurin inhibitors are notably valuable for maintenance therapy, given the long-term side effects associated with topical corticosteroids, although their use is off-label [2].

Patient satisfaction and adherence to the above-mentioned topical treatment options remain low, justifying a demand for alternative therapies. The primary reasons for inadequate adherence to treatment include reduced effectiveness, unsatisfactory cosmetic outcomes, the time-consuming nature of treatment application, and suboptimal doctor-patient relationships [114]. There is a critical need for new molecules that not only demonstrate improved efficacy but also ensure long-term safety. Moreover, these molecules should be formulated in convenient vehicles to enhance patient adherence. Recent advances in understanding the pathogenesis of psoriasis have led to the widespread use of highly effective systemic treatments, and there is a growing focus on emerging topical treatments targeting new therapeutic pathways (Table 2) [2,115].

### 4.1. Aryl Hydrocarbon Receptor (AhR) Modulators

The Aryl hydrocarbon receptor (AhR) is a ligand-dependent transcription factor that regulates gene expression in immune and epithelial cells, including keratinocytes [116]. AhR participates in multiple cellular processes in healthy skin, such as keratinocyte differentiation, skin barrier function and pigmentation, responses to oxidative stress, and the skin immune network, facilitating the terminal differentiation of Th17 and Th22 lymphocytes [117,118].

#### Topical Tapinarof/Benvitimod

Tapinarof, initially known as WBI-1001 and also known as benvitimod and GSK-2894512, is a nonsteroidal anti-inflammatory topical compound with the capability to inhibit the differentiation of Th17 cells and reduce the expression of cytokines, including IL-17, IL-22, and IL-23, through AhR activation. It also regulates protein expression to promote skin barrier normalization [119]. Tapinarof has been evaluated in phase II and phase III clinical trials. A phase IIa trial (NCT01098721) conducted in 61 patients initially showed a significant improvement in disease extension, with almost 80% of the affected body surface being cleared at week 12, compared with patients receiving placebo, who presented worsening of the affected areas [120]. A few years later, a randomized phase IIb trial (NCT02564042) evaluated the efficacy of tapinarof in individuals with mild-to-moderate plaque psoriasis compared with a vehicle lacking an active ingredient [121,122]. Patients were randomized to receive tapinarof at concentrations of 1% BID, 1% QD, 0.5% BID, and 0.5% QD. These groups were compared with two control groups that applied the vehicle cream either twice or once daily. After 12 weeks of follow-up, the groups using tapinarof cream exhibited significantly higher PGA response rates compared with those using the vehicle. Individuals treated with tapinarof cream 1% demonstrated superior response rates than those receiving the 0.5% formulation, and twice-daily application only marginally increased efficacy; thus, once-daily application might be equally effective, enhancing adherence. Tapinarof cream was relatively well tolerated, the most frequently reported side effects being folliculitis and contact dermatitis; they resulted in permanent discontinuation of treatment in 10% of tapinarof-treated patients, with contact dermatitis being the most prevalent cause.

Subsequently, two phase III double-blind randomized trials (PSOARING 1 [NCT03956355] and 2 [NCT03983980]) evaluated the tapinar 1% cream QD compared with placebo cream in 1000 psoriasis patients [123]. A Physician Global Assessment (PGA) score of 0 or 1 was achieved by 35.4% and 40.2% of individuals treated with tapinarof, respectively, compared with 6.0% and 6.3% in the vehicle group. Moreover, 36.1% and 47.6% of the tapinarof-treated participants reached PASI75 responses, in contrast to 10.2% and 6.9% in the vehicle group. Again, the safety profile was generally favorable, although localized adverse effects such as folliculitis and contact dermatitis were reported; folliculitis, predominantly mild to moderate in severity, was documented in up to 20.6% of participants in the tapinarof-treated groups.

Following the completion of the 12-week treatment, eligible subjects (n = 763) were enrolled in an extended 40-week long-term safety and efficacy study (PSOARING 3, NCT04053387) involving the application of 1% tapinarof cream, followed by a subsequent 4-week follow-up period [124]. A total of 40.9% of patients attained complete disease clearance (PGA 0), and among those initially presenting with PGA ≥ 2, 58.2% achieved a PGA score of 0 or 1. In patients who reached a PGA score of 0, the average off-therapy remission duration was 130.1 days. The most common AEs reported were folliculitis (22.7%), contact dermatitis (5.5%), and upper respiratory tract infections (4.7%).

In May 2022, tapinarof cream 1% (Vtama^®^) was approved by the FDA for the treatment of plaque psoriasis in adults, thus becoming the first topical novel chemical treatment launched for psoriasis in the US in the last 25 years [125]. A phase III clinical trial (NCT05172726) is currently evaluating the efficacy and safety of tapinarof in pediatric patients with psoriasis; two phase IV trials are studying its application in special areas such as the head and neck area (NCT05789576) and intertriginous areas (NCT05680740); and another one is assessing its potential combination with biologic therapy (NCT06103695).

A formulation of tapinarof in a different excipient, requiring twice-daily application (Benvitimod 1%) has been studied in a Chinese phase III clinical trial involving 686 patients over 12 weeks, with calcipotriol 0.005% ointment and vehicle as comparators [126]. PGA score of 0 or 1 was achieved by 66.3% of patients treated with benvitimod 1% and 63.9% of those using calcipotriol, superior in both cases to the response rate in the vehicle group (33.5%), but improvement in PGA grade from the baseline was not included as an endpoint, which might result in an exaggerated estimation of improvement. PASI75 response was achieved by a significantly higher percentage of patients treated with benvitimod (50.4%) compared with those treated with calcipotriol (38.5%) or placebo (13.9%). Cutaneous adverse effects were noted in nearly 50% of the benvitimod-treated group, predominantly involving pruritus (found in 21.2% of patients, compared with 10.1% of patients in the calcipotriol group and 12.1% of patients in the placebo group), contact dermatitis, and folliculitis [126]. Subsequent monitoring of 59 patients showed sustained remission up to week 52 in 29 patients (49%), while the remaining patients experienced recurrence on average at 36 weeks [126]. In 2019, Benvitimod 2% was approved for its use in mild-moderate plaque psoriasis in China [127]. A phase IV clinical trial is currently being conducted to evaluate benvitimod cream in adults with psoriasis (NCT05064748), but its results are yet to be posted.

### 4.2. Phosphodiesterase Type-4 (PDE-4) Inhibitors

Elevated phosphodiesterase type 4 (PDE4) activity has been documented in psoriatic skin [128]. PDE4 is an enzyme prominently expressed in immune cells, where it participates in the breakdown of cyclic adenosine monophosphate (cAMP), a crucial intracellular messenger in various signal transduction pathways [129]. Among other roles, cAMP activation of protein kinase A is essential for triggering the production of anti-inflammatory cytokines via phosphorylation of cAMP-responsive element binding protein and activation of transcription factor 1 [129]. Protein kinase A inhibits transcription factors responsible for synthesizing proinflammatory cytokines, such as B-cell lymphoma 6 (Bcl-6) protein and nuclear factor kappa-light-chain-enhancer of activated B cells (NF-κB) [129]. The overexpression of PDE4 in psoriatic skin favors the synthesis of proinflammatory cytokines and the proliferation of immune cells; conversely, suppression of PDE-4 elevates cAMP levels, resulting in reduced expression of cytokines that are crucial in the pathophysiology of psoriasis, such as TNF-α, IFN-γ, IL-17, and IL-23 [128]. Oral PDE-4 inhibitors, such as apremilast and roflumilast, are approved or have been used to treat moderate to severe psoriasis [130]. Increased local availability, minimal absorption, and the potential for adverse effects provide the rationale for the development of topical formulations.

#### 4.2.1. Topical Roflumilast

Roflumilast cream 0.3% QD exhibited both efficacy and good tolerability in a phase IIb trial involving patients with psoriasis (NCT03638258) [131]. During 2020, two twin phase III randomized, double-blind, controlled, multicenter trials (DERMIS-1 [n  =  439, NCT04211363] and DERMIS-2 [n  =  442, NCT04211389]) were conducted [132]. These trials investigated the efficacy of roflumilast cream at 0.3% when applied QD for 8 weeks in patients with plaque psoriasis. Participants were randomly assigned in a 2:1 ratio to receive either roflumilast cream (0.3%) or vehicle cream. In the DERMIS-1 trial, the success rate based on the Investigator Global Assessment (IGA) was 42.4% with roflumilast 0.3% cream, significantly higher compared with 6.1% observed with the vehicle. Similarly, in the DERMIS-2 trial, the IGA success rate was 37.5% for roflumilast 0.3% cream, contrasting with 6.9% for the vehicle. The application of roflumilast cream was associated with a minimal occurrence of adverse effects at the application site and was well tolerated; among the 881 participants, 1% discontinued treatment with roflumilast cream due to adverse reactions, compared with 1.3% of those treated with the vehicle. The most frequent adverse reaction leading to discontinuation of roflumilast was urticaria at the application site (0.3%) [132].

During a 52-week open-label safety study, sustained efficacy in managing chronic plaque psoriasis was observed alongside the absence of new safety concerns associated with long-term treatment [132]. Success rates in treating both plaque psoriasis and intertriginous psoriasis mirrored those observed in the 8-week assessment. By week 52, 45% of patients in the treatment group had achieved an IGA rating of “clear” or “almost clear.”

In July 2022, the FDA granted approval for the application of topical roflumilast cream 0.3% (Zoryve^®^) as a treatment for plaque psoriasis, which included intertriginous areas, for patients aged 12 years and older [133]. More recently, in October 2023, topical roflumilast was also approved by the FDA for children aged 6 to 11 [134]. The expanded approval was granted based on a 4-week study called Maximal Usage Systemic Exposure (MUSE) (NCT04655313), involving children aged 6 to 11 with plaque psoriasis. The pharmacokinetic, safety, tolerability, and efficacy data of the study aligned generally with results from the pivotal phase III trials, DERMIS-1 and DERMIS-2, in adults. Future FDA reviews will include findings from a second MUSE study encompassing children aged 2 to 5 and ongoing data from an open-label extension study (NCT04746911) assessing the long-term safety of roflumilast cream 0.3% in individuals aged 2 years and older with plaque psoriasis.

#### 4.2.2. Topical Crisaborole

Crisaborole (AN2728) is a PDE-4 inhibitor already approved for the topical treatment of atopic dermatitis [135]. During its early development phase, topical crisaborole was also investigated for treatment of plaque psoriasis in a 12-week randomized, double-blinded, vehicle-controlled study (NCT01029405), employing a split-body design where subjects acted as their controls. Crisaborole 0.5% or 2% ointment or vehicle were applied QD or BID. The Crisaborole 2% ointment BID regimen displayed high efficacy with good tolerance and showed no safety concerns. However, further development for its use in psoriasis did not progress, and the outcomes of this study were never published. Additionally, other studies supporting the efficacy of topical crisaborole in plaque psoriasis also remain unpublished [135].

More recently, a small randomized controlled trial (IRB-17-02106) involving 21 patients from a single tertiary care center evaluated the efficacy of crisaborole 2% ointment BID compared with a vehicle in treating intertriginous, anogenital, and facial psoriasis [136]. After 4 weeks, significant improvement was observed in erythema, plaque elevation/induration, and scaling in the crisaborole group compared with the vehicle group. Upon completion of 8 weeks’ treatment, more than 70% of participants achieved clearance of the lesions, and no adverse skin reactions were reported [136].

Despite promising outcomes in small-scale studies, additional exploration of topical crisaborole application in psoriasis has not been conducted. Although topical crisaborole has not obtained regulatory approval for treating psoriasis, its off-label use has been documented [137,138,139].

### 4.3. Janus Kinase (Jak) Inhibitors

As previously mentioned, multiple cytokines implicated in the development of psoriasis use the Jak/STAT pathway for signaling, and targeted inhibition of Jaks diminishes keratinocyte proliferation and mitigates inflammation, providing the basis for studies of the therapeutic potential of their topical administration.

#### 4.3.1. Topical Tofacitinib

Tofacitinib selectively blocks Jak1 and Jak3 and has been under investigation as an oral therapy for psoriasis and psoriatic arthritis [140]. Despite favorable results from two phase III trials involving patients with psoriasis, the FDA finally refrained from approving this medication for psoriasis due to the unfavorable balance between safety and efficacy [15]. Subsequently, tofacitinib has been assessed as a topical treatment for psoriasis in phase II studies [141,142].

A randomized, double-blind, vehicle-controlled phase IIa trial assessed the efficacy and safety of tofacitinib 2% in two ointment formulations (NCT01246583) [141]. Over a 4-week period, 71 individuals with mild-to-moderate psoriasis were administered the designated ointment BID on a specific plaque. When compared with the placebo group, only one of the formulations reached statistical significance in the improvement of the targeted plaque severity score, with modest improvement and limited clinical impact. Although systemic concentrations of tofacitinib were detectable when applied to an area covering approximately 1.5% of the body surface, these levels were regarded as very low and safe [141]. Topical tofacitinib was well tolerated, and no patients discontinued treatment due to side effects.

A phase IIb randomized trial (NCT01831466) involving 435 patients was conducted to investigate the effects of tofacitinib 1% and 2% ointments when applied QD and BID for 12 weeks [142]. By week 8, PGA response, defined as achieving a state of clear (0) or almost clear (1) with a ≥2 grade improvement from baseline, was observed in 18.6% of patients treated with tofacitinib 2% QD and 22.5% for tofacitinib 2% BID, compared with 8.1% and 11.3% for the respective vehicles. However, this modest improvement reached a plateau after week 8, and by week 12, there were no significant differences between groups. Due to the modest efficacy outcomes, further research on topical tofacitinib was discontinued.

#### 4.3.2. Topical Ruxolitinib

Ruxolitinib is a selective inhibitor of Jak1 and Jak2 [140]. The topical formulation of ruxolitinib is currently undergoing evaluation for several dermatological conditions, including atopic dermatitis, vitiligo, and alopecia areata [143]. In the context of psoriasis, initial studies conducted in mice demonstrated that topical application of ruxolitinib resulted in decreased lymphocytic infiltration, keratinocyte proliferation, and acanthosis, prompting further investigations [140]. Two phase II clinical trials have evaluated different concentrations of topical ruxolitinib in patients with plaque psoriasis [144,145].

The first phase II double-blind study assessed the efficacy of ruxolitinib cream in 29 patients with active plaque psoriasis covering less than 20% of the body surface area (NCT00820950) [144]. Patients were randomly assigned to receive 0.5% or 1.0% ruxolitinib cream QD, 1.5% ruxolitinib cream BID, vehicle QD or BID, 0.005% calcipotriene cream BID, or 0.05% betamethasone dipropionate cream BID. For each participant, two similar psoriatic plaques with similar lesion scores were selected: one was treated with ruxolitinib and the other with the respective comparator based on the assigned cohort. After 4 weeks, both the 1% and 1.5% ruxolitinib creams exhibited improvements in lesion thickness, erythema, and scaling and reduced the lesion area in comparison to the placebo, achieving a reduction in the lesion severity score of more than 50% [144]. Interestingly, the 1.5% formulation of ruxolitinib displayed slightly superior performance compared with calcipotriene. Mild adverse effects such as stinging and itching at the application site were reported equally in lesions treated with the vehicle.

Another 4-week phase II open-label multicenter study (NCT00617994) comprising 25 patients with active psoriasis covering 2–20% of the body surface area was designed to assess the safety and efficacy of topical ruxolitinib applied at varying concentrations and body surface area percentages [145]. Patients applied either 1.0% or 1.5% ruxolitinib cream QD or BID for 4 weeks. Across all cohorts, both lesion severity scores and lesion areas were reduced approximately by 50%. All cohorts demonstrated an improvement in the PGA score, which was most notable in the groups treated with 1.5% ruxolitinib BID. However, no subsequent studies have been conducted on this drug for treating psoriasis.

#### 4.3.3. Topical Brepocitinib

As previously mentioned, although it was initially explored as an oral medication in a phase II study that showed promising efficacy, safety concerns prevent its further development. The investigation was then focused on brepocitinib as a topical option. Results from a phase IIb study involving 344 patients have been recently published (NCT03850483) [146]. This multicenter, randomized, double-blind trial was carried out in two phases. During stage 1, participants were administered one of eight treatments for a duration of 12 weeks: brepocitinib 0.1% QD, 0.3% QD or BID, 1.0% QD or BID, 3.0% QD, or vehicle QD or BID. In stage 2, participants were given brepocitinib 3.0% BID or vehicle BID.

At week 12, topical administration of brepocitinib did not result in significant improvement compared with vehicle controls concerning both primary and key secondary efficacy endpoints (change in PASI score from baseline, PGA 0 or 1 with an improvement of ≥2 points from baseline) across any dosage groups. As regards safety, topical brepocitinib was well tolerated, with AEs occurring at similar frequencies across all groups [146].

### 4.4. Potential Future Targets for Topical Therapy

#### 4.4.1. Topical RORγ Inverse Agonists

Various research groups have identified synthetic compounds that target RORγ through different pathways. These ligands can induce changes in RORγ, either (i) decreasing the recruitment of coactivator proteins (inverse agonists), (ii) enhancing coactivator protein recruitment (agonists), or (iii) having no impact on basal transcriptional activity (silent ligands/neutral antagonists).

GSK2981278 is a potent inverse agonist targeting RORγ [147]. Through its action on this receptor, GSK2981278 modulates the principal transcription pathways linked to Th17 cell differentiation and expression. Preclinical studies have demonstrated that GSK2981278 notably hinders the production of Th17 cytokines in both in vitro experiments and human tissue-based systems [147,148].

A phase I randomized, double-blind clinical trial (NCT02548052) evaluated the safety, tolerability, and efficacy of GSK2981278 ointment through repetitive administrations during 19 days across six distinct test regions (each approximately 1.1 cm^2^) located within the psoriatic plaques of 15 participants [149]. The trial evaluated six topical formulations of GSK2981278 ointment in varying concentrations (0.03%, 0.1%, 0.8%, or 4%), a vehicle, and betamethasone valerate 0.1% cream as a comparative positive control. By the end of the 19-day period, only the positive control exhibited a reduction in infiltrate thickness, highlighting the absence of such an effect in other treatment groups. The absence of effect in this investigation could be attributed to the brevity of the treatment duration, a limited area of application, or the possibility that targeting RORγ might not be a feasible approach for topically managing psoriasis if the disease’s pathogenesis entails systemic activity.

Another RORγt inhibitor, S18-000003, has shown a significant decrease in skin inflammation when applied topically in a mouse model of psoriasis, with a low risk of thymus-related side-effects [150]. Similarly, compound 1295-273 has recently been identified to have high activity as a RORγ inhibitor in vitro [151]. However, to date, no further research has been conducted with these molecules in humans.

#### 4.4.2. Interleukin-2 (IL-2) Inhibitors

IL-2 plays a role in regulating T-cell activation and proliferation by acting through an inducible T-cell kinase. BMS-509744 is a small molecule that inhibits IL-2-inducible T-cell kinase. Preclinical studies have explored the effects of topically applied BMS-509744 using an imiquimod-induced lesion mouse model, with promising outcomes, including reduced lesion thickness, diminished inflammatory cell infiltration, and lower messenger RNA levels of Th17-related cytokines (IL-17A, IL-17F, and IL-22) [152].

#### 4.4.3. RNA Modulation

Short noncoding RNA sequences known as microRNAs play an important role in gene expression regulation, controlling the proliferation and differentiation of keratinocytes and T-cells [153]. Variations in miRNA expression levels have been described in subjects with psoriasis when compared with healthy individuals, including both downregulation and upregulation of different miRNA subtypes [153,154,155].

Understanding the behavior of miRNAs could pave the way for their potential modulation. Research has highlighted the feasibility of topical delivery using different carriers. For instance, microRNA-210 demonstrated heightened expression in psoriasis patients and psoriasis-like mouse models [156]. Based on this knowledge, a study has investigated the topical application of a nanocarrier gel containing miRNA-210 antisense in a mouse model. The intervention notably reduced miRNA-210 expression in skin lesions and T cells, leading to improvements in erythema, scaling, acanthosis, and inflammatory infiltrates. Further investigations seem warranted in this area [157].

#### 4.4.4. Amygdalin Analogue

Amygdalin analogues are derivatives of a naturally occurring cyanogenic glycoside found in the kernels and seeds of plants of the Prunus genus (and other food plants) that are purported to exhibit anti-inflammatory properties. Laetrile, a synthetic form of amygdalin, gained attention in the 1970s as an alternative cancer treatment, although its use was not supported by scientific evidence [158]. In addition, consumption of high doses may lead to potential toxicity due to the release of cyanide during its metabolization process [158]. The FDA banned the sale of laetrile as a treatment for cancer in 1980 due to safety concerns and a lack of proven effectiveness [158].

A recent study assessed the effectiveness of topical FIB-116, an amygdalin analogue, on immune-deficient mice with psoriasis xenografts [159]. Over a 15-day period, mice treated with FIB-116 showed improvements in lesion severity and a decrease in psoriasis-like histological features compared with untreated or vehicle-treated mice. The anti-inflammatory effect of amygdalin analogues is thought to be mediated by a decrease in thymic stromal lymphopoietin (*TSLP*) gene expression. TSLP, an inflammatory cytokine, is notably abundant in the epidermis of psoriasis patients and collaborates with the T-cell-derived CD40 ligand to stimulate dendritic cells, fostering the production of IL-23 [160]. FIB-116 exhibited the potential to decrease certain cytokines linked to psoriasis both locally in the skin (IL-17α, TNF-α, and IFN-γ) and systemically in mouse serum (IL-17α, IL-6) [159]. There are no ongoing clinical trials exploring the potential clinical application of FIB-116.

## 5. Discussion

According to existing evidence, the efficacy and safety of oral therapies need significant enhancement to match those of the most recently introduced biologics. Results of tofacitinib were similar to those of one of the first available biologics, etanercept, which has become superseded in the treatment of psoriasis; according to the risk ratios (RRs) of achieving PASI90 response at 8 to 24 weeks, etanercept is numerically superior to ciclosporin (RR 1.51, 95% Confidence Interval [CI] 0.28–8.14), methotrexate (RR 1.53, 95%CI 0.31–7.60), and apremilast (RR 1.39, 95%CI 0.81–2.37), and significantly superior to fumarates (RR 2.45, 95%CI 1.10–5.42) according to the most recent Cochrane meta-analysis [161]. Most oral agents in development have only undergone phase II trials and may not progress further due to their limited efficacy [162].

Deucravacitinib, the first allosteric Tyk2 inhibitor, was approved for treating moderate to severe psoriasis by the FDA in September 2022 and soon later by the EMA and the Japanese regulatory agency, among others. Its short-term efficacy is similar to those of adalimumab (RR 0.81, 95%CI 0.11–5.70) and certolizumab (1.05, 95%CI 0.15–7.57) and numerically superior to etanercept (RR 1.31, 95%CI 0.19–9.29), according to the Cochrane meta-analysis [161]. As it enters real-world usage, deucravacitinib will provide valuable data on its efficacy and safety in psoriasis treatment, defining the role of different Jak inhibitors in the competitive psoriasis treatment landscape.

Among Jak inhibitors, allosteric Tyk2 inhibitors may be the most promising class for oral treatment of moderate to severe psoriasis, with the best efficacy/safety ratio and negligible changes in laboratory parameters. The safety issues of non-selective Jak inhibitors like tofacitinib have prevented their approval for psoriasis [163], but tofacitinib became the first Jak inhibitor approved for treating psoriatic arthritis in 2017 [11], and upadacitinib has been recently approved by the FDA and the EMA for psoriatic arthritis and other indications [164].

Numerous oral treatments employing distinct mechanisms, including A3AR agonists, HSP90 inhibitors, ROCK-2 inhibitors, oral IL-23 inhibitors, oral IL-17 inhibitors, oral TNF inhibitors, PD4 inhibitors (orismilast), and various Tyk2 inhibitors, are presently undergoing clinical trials. Among them, oral IL-23 and IL-17 inhibitors are the most likely candidates to become viable options.

Much-needed real-world data are starting to be gathered with deucravacitinib at the approved dose of 6 m QD, but there is a shortage of head-to-head trials comparing oral medications, with none involving a biologic as an active comparator to date. Indirect comparisons, including meta-analyses, undoubtedly contribute to addressing this matter, but active comparators should be the norm for clinical trials when there are well-defined therapeutic alternatives.

A meta-analysis of the randomized clinical trials evaluating tofacitinib, peficitinib, solcitinib, baricitinib, abrocitinib, and deucravacitinib for treating moderate to severe psoriasis was published in 2022 [165]. Overall, all Jak inhibitors were more likely to achieve a PASI75 response compared with the placebo at both 8 and 12 weeks. Tofacitinib 15 mg BID exhibited the highest probability of attaining PASI75 at both time points, followed by tofacitinib 10 mg BID and deucravacitinib 12 mg QD. In terms of safety, all Jak inhibitors, except for deucravacitinib 6 mg BID and deucravacitinib 12 mg BID, were non-inferior to placebo. In 2023, another network meta-analysis, including 13 randomized clinical trials involving 5274 patients, compared the efficacy and safety of Tyk2 and PDE4 inhibitors in treating moderate to severe plaque psoriasis [166]. Estimated PASI and PGA response rates of deucravacitinib at all doses (excluding 3 mg QOD), ropsacitinib (200 and 400 mg QD), and apremilast (20 and 30 mg BID) were greater than placebo. Furthermore, deucravacitinib (3 mg BID, 6 mg QD, 6 mg BID, and 12 mg QD) and ropsacitinib (400 mg QD) were estimated to be more efficacious than apremilast (30 mg BID). Regarding safety, neither deucravacitinib (at any dose) nor ropsacitinib had a higher occurrence of AEs compared with apremilast (30 mg BID).

Another meta-analysis by Armstrong et al. compared deucravacitinib with other relevant systemic treatments, including biologics and non-biologics, based on the results of 47 randomized clinical trials [46]. According to their results, deucravacitinib was more likely to achieve a PASI 75 response compared with both apremilast and methotrexate consistently across all time intervals. The estimated short-term PASI 75 response rate for deucravacitinib was 54.1%, comparable to that of first-generation biologics such as etanercept, whereas the long-term estimated PASI 75 response rate (65.9%) was similar to those of adalimumab and ustekinumab.

Psoriasis is a chronic inflammatory condition that frequently requires prolonged maintenance treatments due to its relapsing nature upon discontinuation of treatments. Topical therapies are necessary not only for patients with mild psoriasis (namely, limited in extension and amenable to topical treatment) in whom the risk of systemic therapies might exceed the benefit, but also for patients with moderate to severe psoriasis that may require a combination of systemic and topical therapies for complete resolution [167].

Topical corticosteroids, either alone or combined with vitamin D analogues, effectively treat localized lesions of psoriasis, but their long-term use is constrained by potential side effects [140]. Nevertheless, they remain the topical therapy of choice, and ongoing advancements in new formulations [110,111,167] have attempted to improve patient adherence. For over 25 years, the FDA has not approved a topical treatment with a new mechanism of action. Recent advances in our knowledge of psoriasis pathogenesis have led to the development of targeted topical molecules, mostly focused on intracellular signaling pathways such as AhR, PDE-4, and Jak-STAT.

Topical tapinarof, an AhR modulator, and roflumilast, a PDE-4 inhibitor, have exhibited favorable efficacy and safety profiles in extensive phase III trials, and both have been approved by the FDA for the topical treatment of plaque psoriasis in adults and, in the case of roflumilast, in children aged 6 and over.

Tapinarof cream 1% has demonstrated favorable efficacy outcomes when applied QD. The primary adverse reactions reported were folliculitis and contact dermatitis, but these were primarily mild-to-moderate, with no occurrences of severe events. The appearance of folliculitis may limit the applicability of tapinarof in intertriginous areas.

Topical roflumilast cream 0.3% QD also presented favorable efficacy and safety outcomes after 8 weeks of treatment and is approved for patients with psoriasis 6 years of age and older. Positive results were also obtained in patients with intertriginous involvement [131]. A 24-week extension study is presently ongoing to evaluate its safety over an extended period (NCT04286607). Furthermore, it has also been approved by the FDA for the treatment of seborrheic dermatitis in patients 9 years of age and older [168].

Jak-STAT inhibitors were originally evaluated as systemic treatments for psoriasis, demonstrating efficacy but facing safety concerns, which hindered their approval. Efforts to explore the topical application of Jak inhibitors have attained poor success so far. Studies on ruxolitinib and tofacitinib were discontinued due to unsatisfactory efficacy outcomes. Brepocitinib represents the latest addition to this drug class and was expected to obtain better results; nevertheless, recently published phase IIb trials have been disappointing [146].

So far, numerous trials have employed placebos as controls, and comparisons between recently developed topical treatments must be indirect. Recently, a systematic review and Bayesian network meta-analysis using eligible randomized controlled trials involving topical medications (tapinarof, benvitimod, tofacitinib, ruxolitinib, roflumilast, and crisaborole) for plaque psoriasis have been published [169]. Tapinarof 1% BID and tapinarof 1% QD ranked first and second, respectively, in terms of efficacy measured by PGA and PASI75 responses, although with relatively poor safety/tolerance. Differences in efficacy between the 1% QD and 1% BID applications were not significant, and the BID regimen exhibited a less favorable safety profile. As regards topical Jak-STAT inhibitors, all formulations of tofacitinib ointment showed lower efficacy but better safety profiles [169]. Ruxolitinib cream 1% QD exhibited superior PGA response rates but did not differ from the vehicle as regards PASI75 response at both 8 and 12 weeks. Concerning topical PDE-4 inhibitors, the meta-analysis highlighted the efficacy and safety profile of roflumilast, whereas crisaborole ointment did not exhibit a statistically significant difference in efficacy or safety outcomes compared with the vehicle [169].

In a phase I trial, roflumilast 0.5% was compared with betamethasone valerate 0.1% and calcipotriol 0.005% [130]. Topical roflumilast was superior to calcipotriol in efficacy, although not surpassing corticosteroids. Larger studies are needed to assess the approved formulation of roflumilast cream 0.3% against potent corticosteroids, either alone or combined with vitamin D analogues and tapinarof cream 1%.

## 6. Conclusions and Future Directions

Improved understanding of the pathogenesis of psoriasis in the last 30 years has driven the development of various targeted therapies. Biologics have achieved exceptional outcomes, achieving complete clearance in most patients and transforming psoriasis treatment goals. Nevertheless, the need for injections may be challenging for some patients, and efforts are underway to develop novel oral and topical treatments for psoriasis, aiming to overcome the efficacy and safety of oral treatments such as apremilast and older non-biologic drugs. The oral Tyk2 inhibitor deucravacitinib has favorable efficacy and safety profiles, outperforming apremilast at the approved doses. Deucravacitinib provides an oral treatment option with long-term efficacy comparable to certain biologics. Head-to-head comparative studies should be implemented to assess the eventual role of newer oral agents and to disclose the true potential of novel alternatives targeting IL-23 and perhaps IL-17 signaling.

As regards new developments on topical treatment of psoriasis, the efficacy and safety of roflumilast cream 0.3% seem well balanced, whereas the efficacy of topical Jak inhibitors in clinical trials has been disappointing. More extensive and extended prospective studies with active comparators are necessary to assess the long-term efficacy, tolerability, and safety of these novel compounds, examining their cost/benefit and real-world applicability. Future research should prioritize direct comparisons of these treatments with existing options to define their place in the treatment of non-extensive psoriasis amenable to topical treatment.

## Figures and Tables

**Table 1:** Current and emerging oral therapies for psoriasis.

Family	Treatment	Mechanism of Action	Efficacy PASI75	Adverse Events	Future Directions
Jak inhibitors	Tofacitinib	Jak1/Jak3 inhibitor	Week 12: 66.7% Week 52: 79.4%	Mild cytopenia, headache, upper respiratory tract infections, urinary tract infections, and diarrhea	FDA refused its approval for psoriasis treatment in October 2015
	Peficitinib	Jak3 > Jak1 > Jak2 inhibitor	Week 6: 58.8%	Nasopharyngitis, diarrhea, acne, back pain, and contact dermatitis	No ongoing clinical trial
	Solcitinib	Jak1 > Jak2 inhibitor	Week 12: 57%	Headache, nasopharyngitis, nausea, diarrhea, fatigue, and upper abdominal pain	No ongoing clinical trial
	Abrocitinib	Jak1 inhibitor	Week 4: 60%	Nausea, headache, neutropenia, and thrombocytopenia	No ongoing clinical trial
	Baricitinib	Jak1/Jak2 inhibitor	Week 12: 54.1%	Nasopharyngitis	No ongoing clinical trial
	Itacitinib adipate	Jak1 inhibitor	Week 4: 27.7%	Nasopharyngitis, increased AST, headache, and hypertriglyceridemia	No ongoing clinical trial
	Brepocitinib	Tyk2/Jak1 inhibitor	Week 12: 86.2%	Nasopharyngitis, upper respiratory tract infection, and headache	Discontinued
	Ropsacitinib	Tyk2/Jak2 inhibitor	Week 16: 73.2%	Nasopharyngitis, upper respiratory tract infections, and increased blood pressure	No ongoing clinical trial
	Deucravacitinib	Tyk2 inhibitor	Week 12: 75% Week 52: 53%	Nasopharyngitis	Approved
	BMS-986202	Tyk2 inhibitor			Results from phase I trial have not been posted yet
	SAR-20347	Tyk2/Jak1 inhibitor			No trials yet
	TAK-279 (zasocitinib)	Tyk2 inhibitor	Week 12: 68%	Mild or moderate: cytopenias, diarrhea, respiratory tract infections, increased alanine transaminase or creatine kinase serum levels, acne	Two phase III trials are ongoing
	VTX958	Tyk2 inhibitor	No results yet	No serious adverse events	Discontinued
PDE4 inhibitors	Orismilast	PDE4B/PDE4D inhibitor	Week 16: 44.4%	Nausea and diarrhea	Results from phase IIb show inconsistencies, as reported by the FDA
	Mufemilast	PDE4 inhibitor	No results yet		Phase II and phase III trials ongoing
	ME3183	PDE4 inhibitor	Week 16: 61.5%	Nausea, diarrhea, and headache	Phase IIa ongoing
TNF inhibitors	SAR441566	TNF inhibitor	No results yet	No results yet	Results from phase I trial have not been posted yet
IL-17 inhibitors	LY3509754	IL-17 inhibitor	Not reported	Liver toxicity	Trial terminated due to safety concerns related to hepatic function
	DC-806	IL-17 inhibitor	Week 4: 43.7%	Mild or moderate (not specified)	Phase II ongoing
	LEO 153339	IL-17 inhibitor	No results yet	No results yet	Results from phase I trial have not been posted yet
Oral IL-23 inhibitors	JNJ-77242113	Receptor antagonist	Week 16: significant higher PASI75 response compared with placebo	Not reported	Phase I and phase II trials are currently ongoing
RORγT inhibitors	VTP-43472	Inhibition of RORγT	Week 4: 30%	Hepatic toxicity	Discontinued; replaced by VTP-43742
	JTE-451	Inhibition of RORγT	Week 16: 22%	Not reported	No ongoing clinical trial
	AUR1γ01	Inhibition of RORγT	Week 12: 63.3% (phase II); no differences compared with placebo (phase IIb)	Not reported	Development interrupted
	BI 730357	Inhibition of RORγT	Week 12: 30%	Upper respiratory tract infections	No ongoing clinical trial
S1P_1_R antagonist	Ponesimod	Modulator of S1P1R	Week 16: 48.1% Week 28: 77.4%	Dyspnea, liver enzyme abnormalities, and dizziness	No ongoing clinical trial
A3AR agonist	Piclidenoson	Agonist of A3AR	Week 32: 33%	No major adverse events reported	Results from phase III show inconsistencies as reported by the FDA
HSP90 inhibitor	RGRN-305	Inhibition of HSP90	Week 12: 71–94% (PASI50)	Exanthematic reaction	No ongoing clinical trial
ROCK-2 inhibitor	Belumosudil	ROCK2 > ROCK1 inhibitor	Week 12: 16.7%	Diarrhea	Results from phase II have not been posted yet

Abbreviations: PASI: Psoriasis Area and Severity Index; PASI75: improvement in Psoriasis Area and Severity Index (PASI) equal to or greater than 75% with respect to baseline; PASI50: improvement in Psoriasis Area and Severity Index (PASI) equal to or greater than 50% with respect to baseline Jak: Janus Kinase; FDA: Food and Drug Association; PDE4: phosphodiesterase 4; TNF: Tumor Necrosis Factor; IL-17: interleukin 17; IL-23: interleukin 23; RORγT: retinoic acid-related orphan receptors gamma-T; S1P_1_R: Sphingosine-1-phosphate receptor 1; A3AR: A3 adenosine receptor; HSP90: Heat shock protein 90; ROCK-2: Rho-associated protein kinase 2.

**Table 2:** Current and emerging topical therapies for psoriasis.

Family	Treatment	Mechanism of Action	Efficacy Ouctomes	Adverse Events	Discontinuation Due to AEs	Future Directions
Aryl Hydrocarbon Receptor (AhR) Modulators	Tapinarof 1% cream QD	Inhibition of Th17 and Th22 differentiation through AhR activation	Week 12: PASI75 in 36.1% and 47.1% (PSOARING 1 and 2, respectively) Week 40: PGA 0 in 40.9% (PSOARING 3)	- Folliculitis (22.7%) - Contact dermatitis (5.5%) - Upper respiratory tract infections (4.7%)	5.6% and 5.8% (PSOARING 1 and 2, respectively)	Approved by the FDA in May 2022
	Benvitimod 1% cream BID		Week 12: PASI75 in 50.4%	- Pruritus (21,2%) - Folliculitis (10.2%) - Contact dermatitis (8.7%)	Not specified	Approved in China in 2019
Phosphodiesterase Type-4 Inhibitors	Roflumilast 0.3% cream QD	Increase in cAMP levels through inhibition of PDE-4	Week 8: PASI75 in 41.6% and 39.0% (DERMIS-1 and 2, respectively)	- Diarrhea (3.5%) - Hypertension (1.7%) - Headache (1.0%) - Nasopharyngitis (1.7%)	1.7% and 0.3% (DERMIS-1 and 2, respectively)	Approved by the FDA for plaque psoriasis in >12 years in July 2022 and for 6–11 years in October 2023
	Crisaborole 2% ointment BID		Week 4: lesional clearance in 70% of subjects	None	None	No ongoing clinical trials
Jak Inhibitors	Tofacitinib 1% and 2% ointment QD and BID	Jak1 and Jak3 inhibition	Week 8: PGA response * of 18.6% for tofacitinib 2% QD and 22.5% for tofacitinib 2% BID Week 12: no differences to placebo	- Nasopharyngitis (6.5%) - Upper respiratory tract infection (4.9%) - Psoriasis (4.9%)	4.1% (1% tofacitinib QD) 8.6% (2% tofacitinib QD) 1.4% (1% tofacitinib BID) 0% (2% tofacitinib BID)	No ongoing clinical trials
	Ruxolitinib 1% and 2% cream QD and BID	Jak1 and Jak2 inhibition	4 weeks: across all cohorts, both lesion severity scores and lesion areas were reduced approximately by 50%	In total across treatment groups: - Headache (12%) - Seasonal allergy (8%)	Not specified	No ongoing clinical trials
	Brepocitinib	Jak1 and Tyk2 inhibition	Week 12: no significant differences from baseline in least squares mean of the change in PASI scores or PGA response in any treatment group compared with vehicle group	Similar rates of AEs across groups. In total: - Nasopharyngitis (8.2%) - Upper respiratory tract infections (2.9%) - Cough (2.9%)	5.1%	No ongoing clinical trials

* PGA response defined as clear (PGA 0) or almost clear (PGA 1) with a ≥2 grade improvement from baseline. Abbreviations: PASI: Psoriasis Area Severity Index; PASI75: improvement in Psoriasis Area and Severity Index (PASI) equal or greater than 75% with respect to baseline; PGA: Physician Global Assessment; Jak: Janus Kinase; FDA: Food and Drug Association; PDE-4: phosphodiesterase 4; QD: once daily; BID: twice daily.

## Data Availability

Data sharing is not applicable.

## List of Abbreviations

IMID: immune-mediated inflammatory disease; PDE: phosphodiesterase; Jak: Janus kinase; IL: Interleukin; ROR: retinoic acid-related orphan receptors; A3AR: A3 adenosine receptor; HSP: heat shock protein; AhR: Aryl Hydrocarbon Receptor; ROCK: Rho-associated protein kinase; TNF: tumor necrosis factor; STAT: signal transducer and activator of transcription; FDA: Food and Drug Administration; EMA: European Medicines Agency (EMA); PASI: Psoriasis Area and Severity Index; PASI75: improvement in Psoriasis Area and Severity Index (PASI) equal or greater than 75% with respect to baseline; PGA: Physician Global Assessment; DLQI: Dermatology Life Quality Index; BID: twice daily; QD: once daily; AEs: adverse events.
